# Extracting Product Features and Opinion Words Using Pattern Knowledge in Customer Reviews

**DOI:** 10.1155/2013/394758

**Published:** 2013-12-26

**Authors:** Su Su Htay, Khin Thidar Lynn

**Affiliations:** ^1^University of Computer Studies, Mandalay, Myanmar; ^2^Machine and Research Department, University of Computer Studies, Mandalay, Myanmar

## Abstract

Due to the development of e-commerce and web technology, most of online Merchant sites are able to write comments about purchasing products for customer. Customer reviews expressed opinion about products or services which are collectively referred to as customer feedback data. Opinion extraction about products from customer reviews is becoming an interesting area of research and it is motivated to develop an automatic opinion mining application for users. Therefore, efficient method and techniques are needed to extract opinions from reviews. In this paper, we proposed a novel idea to find opinion words or phrases for each feature from customer reviews in an efficient way. Our focus in this paper is to get the patterns of opinion words/phrases about the feature of product from the review text through adjective, adverb, verb, and noun. The extracted features and opinions are useful for generating a meaningful summary that can provide significant informative resource to help the user as well as merchants to track the most suitable choice of product.

## 1. Introduction

Much of the existing research on textual information processing has been focused on mining and retrieval of factual information. Little works had been done on the process of mining opinions until only recently. Automatic extraction of customers' opinions can better benefit both customers and manufacturers. Product review mining can provide effective information that are classifying customer reviews as “recommended” or “not recommended” based on customers' opinions for each product feature. In this cases, customer reviews highlight opinion about product features from various Merchant sites. However, many reviews are so long and only a few sentences contain opinions for product features.

For a popular product, the number of reviews can be in hundreds or even in thousands, which is difficult to be read one by one. Therefore, automatic extraction and summarization of opinion are required for each feature. Actually, when a user expresses opinion for a product, he/she states about the product as a whole or about its features one by one. Feature identification in product is the first step of opinion mining application and opinion words extraction is the second step which is critical to generate a useful summary by classifying polarity of opinion for each feature. Therefore, we have to extract opinion for each feature of a product.

In this paper, we take a written review as input and produce a summary review as output. Given a set of customer reviews of a particular product, we need to perform the following tasks:

1. identifying product feature that customer commented on;
2. extracting opinion words or phrases through adjective, adverb, verb, and noun and determining the orientation;
3. generating the summary.

We use a part-of-speech tagger to identify phrases in the input text that contains adjective or adverb or verb or nouns as opinion phrases. A phrase has a positive semantic orientation when it has good associations (e.g., “awesome camera”) and a negative semantic orientation when it has bad associations (e.g., “low battery”).

The rest of the paper is organized as follows.  Section 2 describes the related work of this paper.  Section 3 elaborates theoretical background for opinion mining.  Section 4 expresses methodology and experiments of the system and Section 5 describes conclusion.

## 2. Related Work

There are several techniques to perform opinion mining tasks. In this section, we discuss others' related works for feature extraction and opinion words extraction. Hu and Liu [1] proposed several methods to analyze customer reviews of format (3). They perform the same tasks of identifying product features on which customers have expressed their opinions and determining whether the opinions are positive or negative. However, their techniques, which are primarily based on unsupervised item sets mining or association rule mining, are only suitable for reviews of formats (3) and (1) to extract product features. Then, frequent item sets of nouns in reviews are likely to be product features while the infrequent ones are less likely to be product features. This work also introduced the idea of using opinion words to find additional (often infrequent) features.

Reviews of these formats usually consist of full sentences. The techniques are not suitable for pros and cons of format (2), which are very brief. Liu et al. [2] presented how to extract product features from “Pros” and “Cons” as type of review format (2). They proposed a supervised pattern mining method to find language patterns to identify product features. They do not need to determine opinion orientations because of using review format (2) indicated by “Pros” and “Cons.”

Hu and Liu [3] proposed a number of techniques based on data mining and natural language processing methods to mine opinion/product features. It is mainly related to text summarization and terminology identification. Their system does not mine product features and their work does not need a training corpus to build a summary. Su et al. [4] proposed a novel mutual reinforcement approach to deal with the feature-level opinion mining problem. Their approach predicted opinions relating to different product features without the explicit appearance of product feature words in reviews. They aim to mine the hidden sentiment link between product features and opinion words and then build the association set.

An approach for mining product feature and opinion based on consideration of syntactic information and semantic information in [5]. The methods acquire relations based on fixed position of words. However, the approaches are not effective for many cases. Turney [6] presented a simple unsupervised learning algorithm for classifying reviews as *recommended *(thumbs up) or *not recommended *(thumbs down). The classification of a review is predicted by the average *semantic orientation *of the phrases in the review that contains adjectives or adverbs. Wu et al. [7] implemented extracting relations between product feature and expressions of opinions. The relation extraction is an important subtask of opinion mining for the relations between more than one product features and different opinion words on each of them.

Wong and Lam [8, 9] employ hidden Markov models and conditional random fields, respectively, as the underlying learning method for extracting product features. Pang et al. [10], Mras and Carroll [11], and Gamon [12] use the data of movie review, customer feedback review, and product review. They use the several statistical feature selection methods and directly apply the machine learning techniques. These experiments show that machine learning techniques only are not well performing on sentiment classification. They show that the presence or absence of word seems to be more indicative of the content rather than the frequency for a word. Zhang and Liu [13] aimed to identify such opinionated noun features. Their involved sentences are also objective sentences but imply positive or negative opinions. They proposed a method to deal with the problem for finding product features which are nouns or noun phrases that are not subjective but objective.

## 3. Mining Opinion for Feature Level

In this paper, we only focus on mining opinions for feature level. This task is not only technically challenging because of the need for natural language processing, but also very useful in practice. For example, businesses always want to find public or consumer opinions about their products and services from the commercial web sites. Potential customers also want to know the opinions of existing users before they use a service or purchase a product. Moreover, opinion mining can also provide valuable information for placing advertisements in commercial web pages. If in a page people express positive opinions or sentiments on a product, it may be a good idea to place an ad of the product. However, if people express negative opinions about the product, it is probably not wise to place an ad of the product. A better idea may be to place an ad of a competitor's product.

There are three main review formats on the Web. Different review formats may need different techniques to perform the opinion extraction task.

Format (1)—pros and cons: The reviewer is asked to describe pros and cons separately.

Format (2)—pros, cons, and detailed review: the reviewer is asked to describe pros and cons separately and also write a detailed review.

Format (3)—free format: the reviewer can write freely, that is, no separation of pros and cons.

For the review formats (1) and (2), opinion (or semantic) orientations (positive or negative) of the features are known because pros and cons are separated. Only product features need to be identified. We concentrate on review format (3) and we need to identify and extract both product features and opinions. This task goes to the sentence level to discover details, that is, what aspects of an object that people liked or disliked. The object could be a product, a service, a topic, an individual, an organization, and so forth. For instance, in a product review sentence, it identifies product features that have been commented on by the reviewer and determines whether the comments are positive or negative. For example, in the sentence, “*The battery life of this camera is too short*,” the comment is on “battery life” of the camera object and the opinion is negative.

Many real-life applications require this level of detailed analysis because, in order to make product improvements, one needs to know what components and/or features of the product are liked and disliked by consumers. Such information is not discovered by sentiment and subjectivity classification [14]. To obtain such detailed aspects, we need to go to the sentence level. Two tasks are apparent.

1. Identifying and extracting features of the product that the reviewers have expressed their opinions on, called product features: for instance, in the sentence “the picture quality of this camera is amazing,” the product feature is “picture quality.”
2. Determining whether the opinions on the features are positive, negative or neutral. In the above sentence, the opinion on the feature “picture quality” is positive.

In the sentence, “the battery life of this camera is too short,” the comment is on the “battery life” and the opinion is negative. A structured summary will also be produced from the mining results.

## 4. Methodology to Find Patterns for Features and Opinions Extraction

The goal of OM is to extract customer feedback data such as opinions on products and present information in the most effective way that serves the chosen objectives. Customers express their opinion in review sentences with single words or phrases. We need to extract these opinion words or phrases in efficient way. Pattern extraction approach is useful for commercial web pages in which customers can be able to write comments about products or services. Let us use an example of the following review sentence: “*The battery life is long.*”

In this sentence, the feature is “*battery life*” and opinion word is “*long*.” Therefore, we first need to identify the feature and opinion from the sentence.

**Figure 1:**
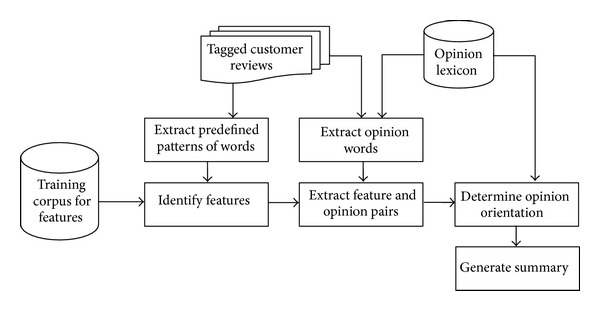
Processing steps for generating feature-based opinion summary.

Figure 1 shows the overall process for generating the results of feature-based opinion summarization. The system input is customer reviews' datasets. We first need to perform POS tagging to parse the sentence and then identify product features and opinion words. The extracted opinion words/phrases are used to determine the opinion orientation which is positive or negative. Finally, we summarize the opinion for each product feature based on their orientations.

In this paper, we focus on feature extraction and opinion word extraction to provide opinion summarization. In feature extraction phase, we need to perform part-of-speech tagging to identify nouns/noun phrases from the reviews that can be product features. Nouns and noun phrases are most likely to be product features.

POS tagging is important as it allow us to generate general language patterns. We use Stanford-POS tagger to parse each sentence and yield the part-of-speech tag of each word (whether the word is a noun, adjective, verb, adverb, etc.) and identify simple noun and verb groups (syntactic chunking), for instance,

*The_DT photo_JJ quality_NN is_VBZ amazing_JJ and_CC i_FW know_VBP i_FW ‘m_VBP going_VBG to_TO have_VB fun_NN with_IN all_PDT the_DT features_NNS._.*

After POS tagging is done, we need to extract features that are nouns or noun phrases using the pattern knowledge (see Table 1). And then, we focus on identifying domain product features that are talked about by customers by using the manually tagged training corpus for domain features.

For opinion words extraction, we used extracted features that are used to find the nearest opinion words with adjective/adverb. To decide the opinion orientation of each sentence, we need to perform three subtasks. First, a set of opinion words (adjectives, as they are normally used to express opinions) is identified. If an adjective appears near a product feature in a sentence, then it is regarded as an opinion word. We can extract opinion words from the review using the extracted features, for instance;

*The strap is horrible and gets in the way of parts the camera you need access to.*

*After nearly 800 pictures I have found that this camera takes incredible pictures.*

*It comes with a rechargeable battery that does not seem to last all that long, especially if you use the flash a lot.*

For the first sentence, the feature, *strap*, is near the opinion word horrible. And in the second example, feature “picture” is close to the opinion word incredible. We found that opinion words/phrases are mainly adjective/adverb that is used to qualify product features with nouns/noun phrases. In this case, we can extract the nearby adjective as opinion word if the sentences contain any features. However, for the third sentence, the feature, battery, cannot be able to extract nearby adjective to meet the opinion word “long.” The nearby adjective “rechargeable” dose not bear opinion for the feature “battery.”

Moreover, both adjective and adverb are good indicators of subjectivity and opinions. Therefore, we need to extract phrases containing adjective, adverb, verb, and noun that imply opinion. We also consider some verbs (like, recommend, prefer, appreciate, dislike, and love) as opinion words. Some adverbs like (not, always, really, never, overall, absolutely, highly, and well) are also considered. Therefore, we extract two or three consecutive words from the POS-tagged review if their tag conforms to any of the patterns. We collect all opinionated phrases of mostly 2/3 words like (adjective, noun), (adjective, noun, noun), (adverb, adjective), (adverb, adjective, noun), (verb, noun), and so forth from the processed POS-tagged review.

The resulting patterns are used to match and identify opinion phrases for new reviews after the POS tagging. However, there are more likely opinion words/phrases in the sentence but they are not extracted by any patterns. From these extracted patterns, most of adjectives or adverbs imply opinion for the nearest nouns/noun phrases.  Table 2 described some examples of opinion phrases.

### 4.1. Dataset of the System

We used annotated customer reviews' data set of 5 products for testing. All the reviews are from commercial web sites such as http://www.amazon.com/ and http://www.epinion.com. Each review consists of review title and detail of review text. The reviews are retagged manually based on our own feature list. Each camera review sentence is attached with the mentioned features and their associated opinion words. Therefore, we only focus on the review sentences that contain opinions for product features, for instance, *“The pictures are absolutely amazing—the camera captures the minutest of details.”*

This sentence will receive the tag: picture [+3]. Words in the brackets are those we found to be associated with the corresponding opinion orientation of feature whether positive or negative (see Table 3).

### 4.2. Experiments

We carried out the experiments using customer reviews of 5 electronic products: two digital cameras, one DVD player, one MP3 player, and one cellular phone. All the reviews are extracted from http://www.amazon.com/. All of them are used as the training data to mine patterns. These patterns are then used to extract product features from test reviews of these products. We now evaluate the proposed automatic technique to see how effective it is in identifying product features and opinions from customer reviews. In this paper, we only verify only product features but we make sentiment orientation of opinion on that features as an ongoing process. The effectiveness of the proposed system has been verified with review set on these five different electronic products. All the results generated by our system are compared with the manually tagged results. We also assess the time saved by semiautomatic tagging over manual tagging. We showed the comparison results with Hu and Liu's approach and our approach is slightly higher than their results in Table 4.

## 5. Conclusion

Most of opinion mining researches use a number of techniques for mining opinion and summarizing opinions based on features in product reviews based on data mining and natural language processing methods. Review text is unstructured and only a portion or some sentences include opinion-oriented words. In product reviews, users write comments about features of products to describe their views according to their experience and observations. The first step of opinion mining in classifying reviews' documents is extracting features and opinion words. Therefore opinion mining system needs only the required sentences to be processed to get knowledge efficiently and effectively. We proposed the ideas to extract patterns of features and/or opinion phrases. We showed results of experiments with extracting pattern knowledge based on linguistic rule. We expected to achieve good results by extracting features and opinion-oriented words from review text with help of adjectives, adverbs, nouns, and verbs. We believe that there is rich potential for future research. For identifying feature, we need to extend both explicit and implicit feature as our future work because both of these features are useful for providing more accurate results in determining the polarity of product/feature before summarizing them, rather than explicit feature only.

## Figures and Tables

**Table 1:** Extracted phrases' patterns.

Pattern	The first word	The second word	The third word
Pattern 1	JJ	NN/NNS	—
Pattern 2	JJ	NN/NNS	NN/NNS
Pattern 3	RB/RBR/RBS	JJ	—
Pattern 4	RB/RBR/RBS	JJ/RB/RBR/RBS	NN/NNS
Pattern 5	RB/RBR/RBS	VBN/VBD	—
Pattern 6	RB/RBR/RBS	RB/RBR/RBS	JJ
Pattern 7	VBN/VBD	NN/NNS	—
Pattern 8	VBN/VBD	RB/RBR/RBS	—

**Table 2:** A few examples of extracted opinionated phrases.

(Adjective, noun)	(low battery), (good memories), (awesome camera), and so forth
(Adjective, noun, noun)	(high quality pictures)
(Adverb, adjective)	(extremely pleased), (very easy), (really annoying), (absolutely amazing), and so forth
(Adverb, adjective, noun)	(very compact camera), (very good pictures), and so forth
(Adverb, verb)	(personally recommend)
(Adverb, adverb, adjective)	(not so bad), and so forth
(Verb, noun)	(recommend camera), (appreciate picture), and so forth
(Verb, adverb)	(perform well)

**Table 3:** Summary of tagged product features for each customer review dataset.

	Number of tagged product features	Number of review sentences
Apex	110	739
Canon	100	597
Creative	180	1716
Nikon	74	346
Nokia	109	546

**Table 4:** The performance comparison of Hu and Liu's approach and our approach for feature extraction.

	Hu and Liu's approach			Our approach		
	Recall	Precision	*F*-measure	Recall	Precision	*F*-measure
Apex	0.797	0.743	0.769	0.970	0.782	0.866
Canon	0.822	0.747	0.788	0.921	0.739	0.820
Creative	0.818	0.692	0.750	0.762	0.696	0.728
Nikon	0.792	0.710	0.749	0.812	0.712	0.759
Nokia	0.761	0.718	0.739	0.821	0.736	0.776
Average	**0.80**	**0.72**	**0.758**	**0.857**	**0.733**	**0.790**
